# Handheld Ultrasound as a Novel Predictive Tool in Atrial Fibrillation: Prediction of Outcomes Following Electrical Cardioversion

**DOI:** 10.2196/cardio.9534

**Published:** 2018-03-08

**Authors:** Devin Kehl, Raymond Zimmer, Madhuri Sudan, Ilan Kedan

**Affiliations:** ^1^ Palo Alto Foundation Medical Group Palo Alto, California, CA United States; ^2^ Cedars Sinai Heart Institute Beverly Hills, CA United States; ^3^ Department of Epidemiology UCLA School of Public Health Los Angeles, CA United States; ^4^ Department of Public Health Aarhus University Aarhus Denmark; ^5^ College of Osteopathic Medicine of the Pacific Western University of Health Sciences Pomona, CA United States

**Keywords:** atrial fibrillation, cardioversion, recurrence, inferior vena cava, hand held ultrasound, point of care

## Abstract

**Background:**

Atrial fibrillation (AF) recurrence after successful direct current cardioversion (CV) is common, and clinical predictors may be useful. We evaluated the risk of early AF recurrence according to inferior vena cava (IVC) measurements by handheld ultrasound (HHU) at the time of CV.

**Objective:**

Assess HHU and objectively obtained measurements acquired at the point of care as potential clinical predictors of future clinical outcomes in patients with AF undergoing CV.

**Methods:**

Maximum IVC diameter (IVCd) and collapsibility with inspiration were measured by the Vscan HHU (General Electric Healthcare Division) in 128 patients immediately before and after successful CV for AF. Patients were followed by chart review for recurrence of AF.

**Results:**

Mean IVCd was 2.16 cm in AF pre-CV and 2.01 cm in sinus rhythm post-CV (*P*<.001). AF recurred within 30 days of CV in 34 of 128 patients (26.6%). Among patients with IVCd <2.1 cm pre-CV and decrease in IVCd post-CV, AF recurrence was 12.1%, compared to 31.6% in patients not meeting these parameters (odds ratio [OR] 0.299, *P*=.04). This association persisted after adjustment for age, ejection fraction <50%, left atrial enlargement, and amiodarone use (adjusted OR 0.185, *P*=.01). Among patients with IVCd post-CV <1.7 cm, AF recurrence was 13.5%, compared to 31.9% in patients not meeting this parameter (OR 0.185, *P*=.01). IVC parameters did not predict AF recurrence at 180 or 365 days.

**Conclusions:**

The presence of a normal IVCd pre-CV that becomes smaller post-CV and the presence of a small IVCd post-CV were each independently associated with reduced likelihood of early, but not late, AF recurrence. HHU assessment of IVCd at the time of CV may be useful to identify patients at low risk of early recurrence of AF after CV.

## Introduction

Atrial fibrillation (AF), one of the most common abnormal rhythms of the heart, is associated with significant morbidity including heart failure and stroke [1]. In addition, up to 90% of patients may be symptomatic from AF [2]. Direct-current electrical cardioversion (CV) to restore sinus rhythm (SR) may be performed to improve symptoms, quality of life, and left ventricular function [3]. However, CV may be immediately unsuccessful in up to 20% of cases, and AF recurs in 60-80% of patients after one year [4]. Reliable prediction of SR maintenance after CV is important in order to weigh the benefits versus potential risks of CV including arrhythmias and thromboembolic complications. Several clinical, echocardiographic, and electrophysiological parameters have been identified to predict SR maintenance after CV, including age [5], left atrial size [4,6,7], right atrial size [8], prolonged duration of AF [9], low ejection fraction [4], mitral valve disease [4,5], or failure to correct the underlying physiologic trigger for AF. More recently, left atrial filling pressure and change in pressure after restoration of SR have been identified as predictors of AF recurrence [10,11]. Nevertheless, AF recurrence after CV remains common.

Assessment of the inferior vena cava (IVC) by ultrasound is a validated, noninvasive technique to assess central venous pressure and guide management in patients with dyspnea, critical illness, and heart failure. Current guidelines published by the American Society of Echocardiography in 2015 support the combined use of maximum IVC diameter (IVCd; > or <2.1 cm) and inspiratory IVC collapsibility (> or <50%) to estimate right atrial pressure (RAP) as low, intermediate, or high [12]. Handheld ultrasound (HHU) has emerged as an effective tool to obtain these measurements in a rapid and reproducible fashion at the point of care. This study had two primary objectives. The first was to assess the dynamic changes in IVC size as assessed by HHU according to the presence of AF, through measurements of the IVC immediately prior to and following CV. The second aim was to assess the relationship between IVC size at the time of CV and the risk of AF recurrence after CV.

## Methods

Consecutive adult patients who were scheduled to undergo inpatient or outpatient direct current CV for AF or atrial flutter (AF/AF) at Cedars-Sinai Medical Center (Los Angeles, CA) were enrolled from January 2016 to May 2017. The longitudinal cardiovascular care for these patients also occurred at Cedars-Sinai Medical Center or affiliated outpatient offices. Patients both with and without a concomitant transesophageal echocardiogram at the time of CV were eligible for inclusion. Patients were excluded from analysis if direct current CV was not performed for any reason, or was unsuccessful in restoring SR. All subjects provided written informed consent. This was an investigator-initiated observational study, and the study protocol was reviewed and approved by the Cedars-Sinai Institutional Review Board.

**Figure 1:**
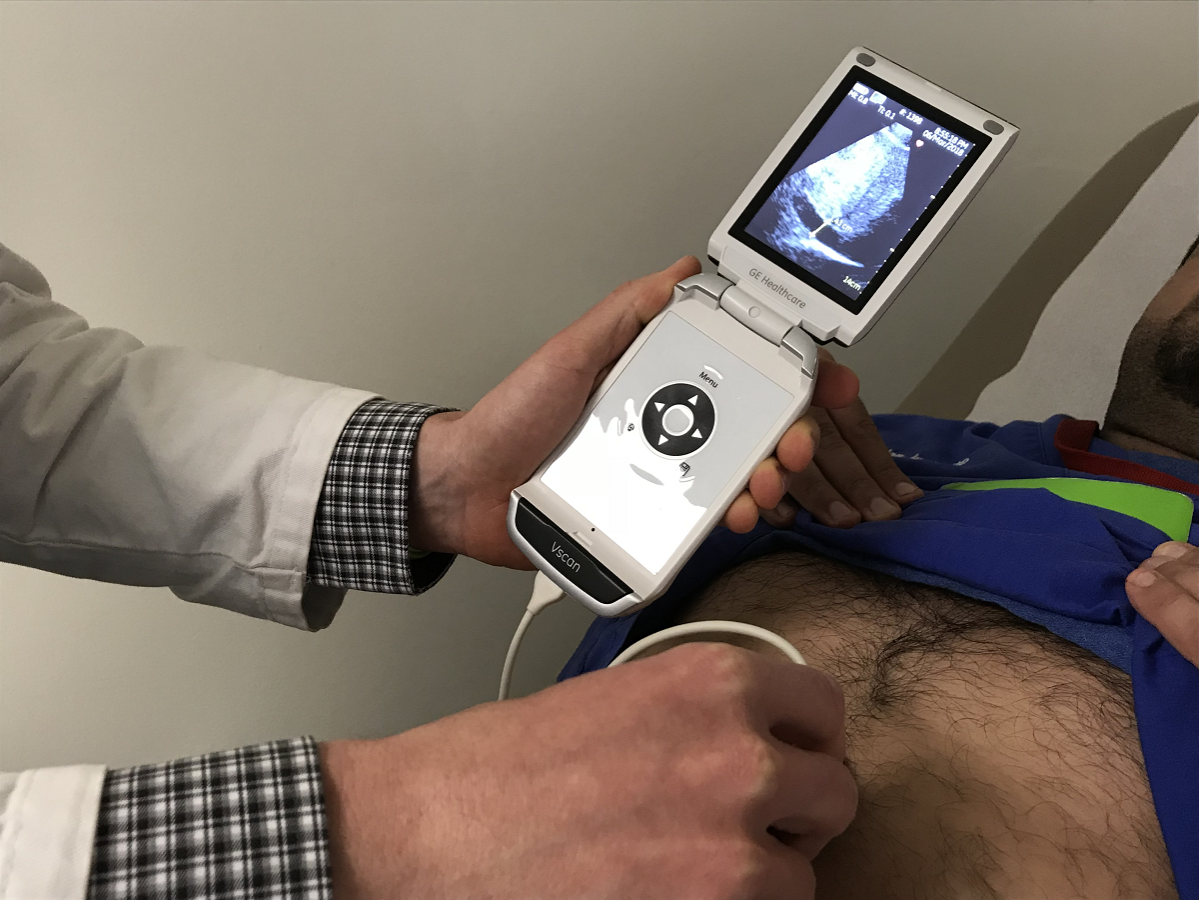
Handheld ultrasound.

Measurements of the IVC were obtained with the patient in a supine position using a HHU device (VScan, General Electric Healthcare Division; Figure 1), during periods of quiet spontaneous respiration. IVCd throughout the respiratory cycle was measured from the subcostal view 1-2 cm caudal to the right atrium/IVC junction with the IVC displayed along its long axis, in accordance with American Society of Echocardiography 2015 published guidelines [12]. Measurements were taken to the nearest one hundredth of a centimeter. A visual estimate of degree of collapsibility (> or <50%) with passive inspiration was also recorded. HHU images used for measurement were saved and uploaded into the Epic-based electronic health record using the image screen capture tool on the Epic-based Haiku smartphone app [13]. Measurements of the IVC were obtained prior to CV (within 20 minutes) with the patient in AF/AF, and repeated within 20 minutes of successful CV with the patient in SR. All patients received deep sedation with propofol for the CV procedure. Sedation was not administered until after the baseline IVC measurement. Intravenous fluid was not added during sedation for any patients. All IVC measurements were obtained by study personnel with level II certification in adult echocardiography as well as special expertise in the use of HHU.

Blood pressure and heart rate were recorded at the time of ultrasound image acquisition both prior to and following CV. Baseline demographics, clinical history, and echocardiographic data were obtained by chart review. The categorization of unspecified, paroxysmal, and persistent AF was defined by chart documentation and was included even though all patients undergoing CV were determined to have persistent AF by study personnel. Echocardiographic data was only included if performed within 6 months prior to the date of CV, and was obtained from review of echocardiogram reports alone. Patients were followed by chart review for up to 365 days for the outcome of recurrence of AF/AF, which was determined by review of electrocardiograms, Holter monitors, and medical documentation.

Descriptive statistical analyses were performed. Data are reported as mean (standard deviation) or total number and percentage, as appropriate. Comparisons of characteristics between groups were made with the Fisher exact test for categorical variables and the Student t-test for continuous variables. All reported *P* values are two-sided. A Kaplan-Meier analysis was performed to compare the freedom from recurrence of AF/AF between groups with differing IVC parameters, and the log-rank test was used to compare groups. Bivariate and multivariate logistic regression analyses were performed for the association between several IVC parameters and recurrence of AF/AF at 30, 90, 180, and 365 days after CV. The multivariable model included adjustment for history of age, ejection fraction <50%, left atrial enlargement (none, mild, moderate, severe), and amiodarone use post-CV factors.

An IVC diameter of 2.1 cm was used as the cut-point in the analysis to remain consistent with published American Society of Echocardiography guidelines used to estimate RAP [12]. A 20% change in IVC diameter following CV was anticipated (1.7 cm) from our study preenrollment power calculation, and was thus used as a cut-point for analyses as well.

## Results

One hundred and fifty-nine patients consented for enrollment in the study. Of these, 11 patients were excluded from the analysis after CV was aborted due to the discovery of (or inability to) exclude left atrial appendage thrombus on a transesophageal echocardiogram performed prior to CV. An additional 15 patients were excluded due to unsuccessful CV. One patient was excluded due to inability to save the HHU measurements of the IVC. Four patients were excluded due to the spontaneous conversion to SR prior to CV. In total, 128 patients were included for analyses.

Baseline demographics of patients included for analyses are summarized in Table 1. The mean age was 67.7 years, and 91 of the 128 patients (71.1%) were male. Thirty patients (30/128, 23.4%) had a history of heart failure with reduced ejection fraction, and 14 patients (14/128, 10.9%) had a history of heart failure with preserved ejection fraction. Twenty-three patients (23/128, 18.0%) had a chart-documented history of persistent AF, with 15 (15/128, 11.7%) having a prior history of ablation and 35 (35/128, 27.3%) having a prior history of CV. Fifty-two patients (52/128, 40.6%) were prescribed amiodarone following CV for maintenance of SR.

Thirty-day follow-up data were available for all 128 patients. One hundred and nineteen patients were followed for at least 90 days, 101 patients were followed for at least 180 days, and 80 patients were followed for 365 days. AF/AF recurrence was seen in 34 patients (34/128, 26.6%) within 30 days, 45 patients (45/128, 37.8%) within 90 days, 44 patients (44/128, 43.6%) within 180 days, and 42 patients (42.128, 52.5%) within 365 days (data not shown). Patients were excluded from the numerator of AF/AF patients and the analysis of study patients if they were not monitored for 90, 180, and 365 days.

Baseline characteristics were compared between patients with and without AF/AF recurrence within 30 days (Table 1). No significant difference was seen between groups with respect to age, gender, body mass index, inpatient status, coronary artery disease, hypertension, diabetes, chronic kidney disease, chronic lung disease, or heart failure. Chart-documented persistent AF was more common among patients with AF/AF recurrence (32.4% vs 12.8%, *P*=.01). Amiodarone use following CV was also more common among patients with recurrence (55.9% vs 35.1%, *P*=.04).

Baseline echocardiographic data were available for most, but not all, patients, and they are summarized in Table 2. Mean ejection fraction was 52.6% overall, and was normal (>55%) in 78 of 121 patients (64.5%). Fifteen patients (15/121, 12.4%) had an ejection fraction <35%. Left atrial enlargement was seen in 97 of 117 patients (82.9%), but severe left atrial enlargement was seen in only 4 patients (4/117, 3.4%). Right atrial enlargement was seen in 54 of 113 patients (47.8%).

Moderate or greater mitral regurgitation was seen in 22 of 118 patients (18.6%). Moderate or greater tricuspid regurgitation was seen in 15 of 109 patients (13.8%). There were no significant differences seen between patients with and without 30-day AF/AF recurrence in ejection fraction, left atrial size, right atrial size, mitral regurgitation, or tricuspid regurgitation.

**Figure 2:**
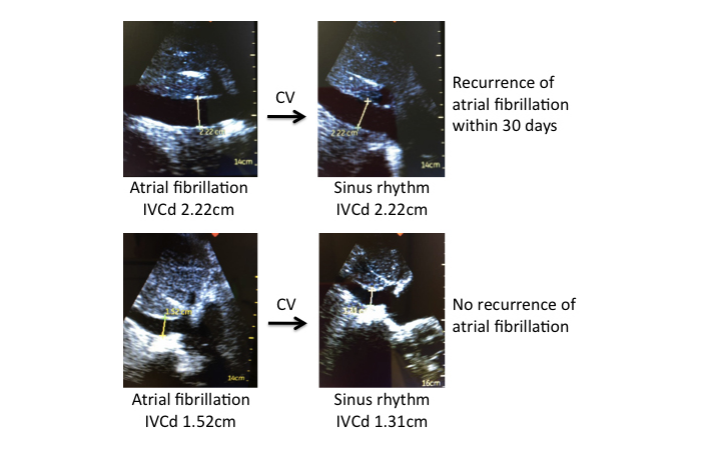
Example measurements of the inferior vena cava by handheld ultrasound.

**Figure 3:**
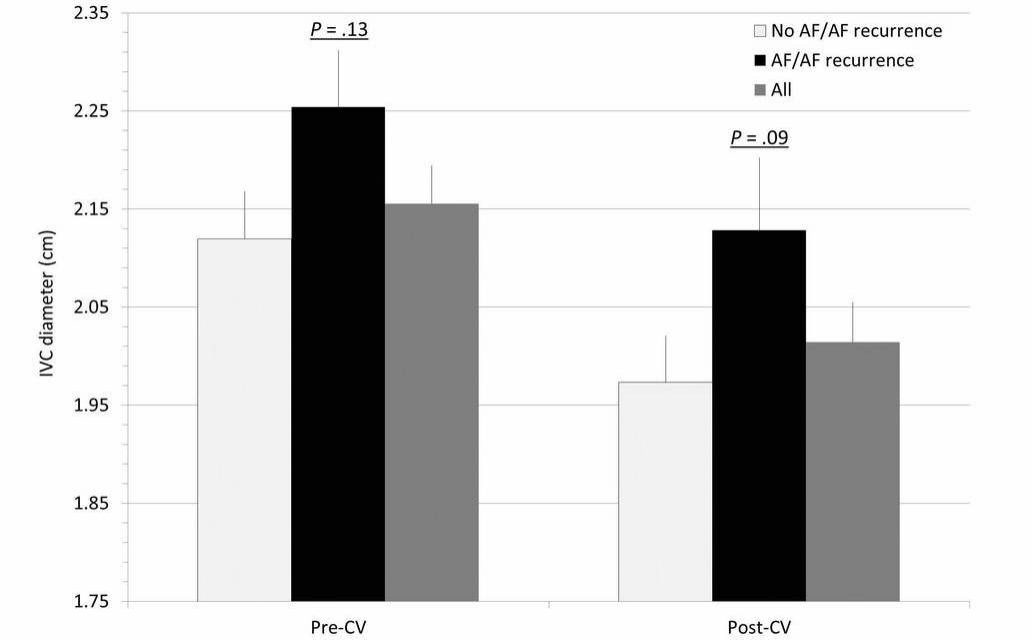
Mean inferior vena cava measurements.

Example IVC measurements pre- and post-CV are shown in Figure 2, and all measurements are summarized in Table 2 and Figure 3. The mean pre-CV IVCd was 2.16 cm, compared to 2.01 cm in the same patients post-CV (*P*<.001). Inspiratory collapsibility >50% was seen in 63 patients (63/128, 49.2%) pre-CV, compared to 93 patients (93/128, 72.7%) post-CV (*P*<.001). RAP was estimated to be high (10-20 mmHg) in 51 patients (51/128, 39.8%), intermediate (5-10 mmHg) in 35 (35/128, 27.3%), and low (0-5 mmHg) in 42 (42/128, 32.8%) prior to CV, and after CV it was estimated to be high in 24 patients (24/128, 18.8%), intermediate in 40 (40/128, 31.3%), and low in 64 (64/128, 50.0%; *P*<.001). Thirty-six patients (36/128, 28.1%) were reclassified to a lower category of RAP following CV, and only 3 patients (3/128, 2.3%) were reclassified to a higher category of RAP following CV. When comparing patients with and without 30-day AF/AF recurrence, there was no significant difference in IVCd pre-CV (2.25 vs 2.12 cm, *P*=.13). However, patients with 30-day AF/AF recurrence were less likely to have inspiratory collapsibility >50% (35.5% vs 54.3%, *P*=.07) and more likely to have a larger IVCd post-CV (2.13 vs 1.97 cm, *P=*.09).

Systolic blood pressure, diastolic blood pressure, and heart rate were significantly higher in AF compared to SR (128.3 vs 105.8 mmHg, *P*<.001; 78.0 vs 65.5 mmHg, *P*<.001; 92.5 vs 67.0 beats per minute, *P*<.001; respectively). No significant correlation was seen between systolic blood pressure, diastolic blood pressure, or heart rate and either pre- or post-CV IVC size. Furthermore, no association was seen between the change in blood pressure or heart rate following CV and the change in IVC size following CV (data not shown).

The combined presence of IVCd <2.1 cm pre-CV and a change in IVCd <0 was associated with a lower rate of AF/AF recurrence at 30 days (12.1% vs 31.6%, unadjusted odds ratio [OR] 0.299, 95% CI 0.096-0.926, *P*=.04; Table 3). This association remained statistically significant (adjusted OR 0.178, 95% CI 0.046-0.682, *P*=.01) in the multivariate model. The combined parameter was similarly associated with a lower rate of AF/AF recurrence at 90 days (unadjusted OR 0.361, 95% CI 0.141-0.924, *P*=.03; adjusted OR 0.265, 95% CI 0.089-0.793, *P*=.02). No significant association was seen with AF/AF recurrence at 180 days or 365 days. Furthermore, the presence of IVCd <1.7 cm post-CV was associated with a lower rate of AF/AF recurrence at 30 days (13.5% vs 31.9%), corresponding to an unadjusted OR of 0.334 (95% CI 0.118-0.946, *P*=.04). This association remained statistically significant in the multivariate model (adjusted OR 0.185, 95% CI 0.050-0.691, *P*=.01).

**Table 1:** Baseline demographics.

Parameter		Total (N=128)	30-day AF/AF recurrence (n=34)	No 30-day AF/AF recurrence (n=94)	*P* value
Age, mean (SD)		67.7 (13.4)	66.9 (13.5)	68.0 (13.5)	.70
Male gender, n (%)		91 (71.1)	26 (76.5)	65 (69.1)	.51
Body mass index (kg/m^2^), mean (SD)		28.4 (6.6)	28.7 (6.1)	28.3 (6.8)	.77
Inpatient, n (%)		47 (36.7)	11 (32.3)	36 (38.3)	.68
Coronary artery disease, n (%)		33 (25.8)	7 (20.6)	26 (27.7)	.49
Hypertension, n (%)		85 (66.4)	27 (79.4)	58 (61.7)	.09
Diabetes mellitus type II, n (%)		21 (16.4)	6 (17.6)	15 (16.0)	.79
Chronic kidney disease, n (%)		29 (22.7)	8 (23.5)	21 (22.3)	1.00
Chronic lung disease, n (%)		11 (8.6)	4 (11.8)	7 (7.4)	.48
Heart failure with reduced ejection fraction, n (%)		30 (23.4)	11 (32.4)	19 (20.2)	.16
Heart failure with preserved ejection fraction, n (%)		14 (10.9)	3 (8.8)	11 (11.7)	.76
**Atrial fibrillation (n=117), n (%)**					.01
	Unspecified	18 (14.1)	1 (2.9)	17 (18.1)	
	Paroxysmal	76 (59.4)	22 (64.7)	54 (57.4)	
	Persistent	23 (18.0)	11 (32.4)	12 (12.8)	
Atrial flutter (any), n (%)		24 (18.8)	4 (11.8)	20 (21.3)	.31
Prior cardioversion, n (%)		35 (27.3)	8 (23.5)	27 (28.7)	.66
Prior ablation, n (%)		15 (11.7)	3 (8.8)	12 (12.8)	.76
Amiodarone (post-CV), n (%)		52 (40.6)	19 (55.9)	33 (35.1)	.04
Any anti-arrhythmic drug (post-CV), n (%)		69 (53.9)	21 (61.8)	48 (51.1)	.32

**Table 2:** Baseline echocardiographic data and IVC parameters pre-CV and post-CV.

Parameter		Total	30-day AF/AF recurrence (n=34)	No 30-day AF/AF recurrence (n=94)	*P* value
**Ejection fraction (n=121)**					
	>55%, n (%)	78 (64.5)	21 (61.8)	57 (65.5)	
	35-54%, n (%)	28 (23.1)	7 (20.6)	21 (24.1)	.34
	<35%, n (%)	15 (12.4)	6 (17.6)	9 (10.3)	
	Mean (SD)	52.6 (14.4)	50.9% (16.8%)	53.3% (13.4%)	.40
**Left atrial enlargement (n=117), n (%)**					.48
	None	20 (17.1)	4 (13.3)	16 (18.4)	
	Mild	64 (54.7)	20 (66.7)	44 (50.6)	
	Moderate	29 (24.8)	5 (16.7)	24 (27.6)	
	Severe	4 (3.4)	1 (3.3)	3 (3.4)	
**Right atrial enlargement (n=113), n (%)**					.16
	None	59 (52.2)	12 (40.0)	47 (56.6)	
	Mild	37 (32.7)	14 (46.7)	23 (27.7)	
	Moderate	14 (12.4)	4 (13.3)	10 (12.0)	
	Severe	3 (2.7)	0 (0)	3 (3.6)	
**Mitral regurgitation (n=118), n (%)**					.33
	None/trace	33 (28.0)	11 (35.5)	22 (25.3)	
	Mild	63 (53.4)	13 (41.9)	50 (57.5)	
	Moderate	20 (16.9)	7 (22.6)	13 (14.9)	
	Severe	2 (1.7)	0 (0)	2 (2.3)	
**Tricuspid regurgitation (n=109), n (%)**					.79
	None/trace	47 (43.1)	13 (44.8)	34 (42.5)	
	Mild	47 (43.1)	13 (44.8)	32 (40.0)	
	Moderate	13 (11.9)	3 (10.3)	10 (12.5)	
	Severe	2 (1.8)	0 (0)	2 (2.5)	
IVCd pre-CV (cm), mean (SD)		2.16 (0.44)	2.25 (0.34)	2.12 (0.47)	.13
IVC collapsibility >50% pre-CV, n (%)		63 (49.2)	12 (35.3)	51 (54.3)	.07
**RAP estimate pre-CV, n (%)**					
	High (10-20 mmHg)	51 (39.8)	18 (52.9)	33 (35.1)	
	Intermediate (5-10 mmHg)	35 (27.3)	9 (26.5)	26 (27.7)	.13
	Low (0-5 mmHg)	42 (32.8)	7 (20.6)	35 (37.2)	
IVCd post-CV (cm), mean (SD)		2.01 (0.46)	2.13 (0.43)	1.97 (0.46)	.09
IVC collapsibility >50% post-CV, n (%)		93 (72.7)	22 (64.7)	71 (75.5)	.26
**RAP estimate post-CV, n (%)**					
	High (10-20 mmHg)	24 (18.8)	7 (20.6)	17 (18.1)	
	Intermediate (5-10 mmHg)	40 (31.3)	16 (47.1)	25 (26.6)	.10
	Low (0-5 mmHg)	64 (50.0)	12 (35.3)	52 (55.3)	
Delta IVCd (cm), mean (SD)		-0.14 (0.27)	-0.13 (0.36)	-0.15 (0.24)	.71
Pre-CV IVCd <2.1 cm, mean (SD)		57 (44.5)	12 (35.3)	45 (47.9)	.23
Pre-CV IVCd <2.1 cm & Delta IVCd <0, mean (SD)		33 (25.8)	4 (11.8)	29 (30.9)	.04
Post-CV IVCd <1.7 cm, mean (SD)		37 (28.9)	5 (14.7)	32 (34.0)	.05

**Table 3:** Risk of AF recurrence by HHU-derived IVC parameters.

Parameter and follow-up (days)		AF/AF recurrence rate (%)		Odds Ratio (95% CI)			
		Parameter present	Parameter absent	Unadjusted	*P* value	Adjusted^a^	*P* value
**IVCd pre-CV <2.1 cm & delta IVCd <0**							
	30	12.1	31.6	0.299 (0.096-0.926)	.04	0.178 (0.046-0.682)	.01
	90	21.9	43.7	0.361 (0.141-0.924)	.03	0.265 (0.089-0.793)	.02
	180	28.0	48.7	0.410 (0.154-1.095)	.08	0.459 (0.155-1.357)	.16
	365	40.9	56.9	0.525 (0.194-1.420)	.20		
**IVCd post-CV <1.7 cm**							
	30	13.5	31.9	0.334 (0.118-0.946)	.04	0.185 (0.050-0.691)	.01
	90	25.0	43.4	0.435 (0.182-1.039)	.06	0.344 (0.124-0.958)	.04
	180	34.5	47.2	0.588 (0.240-1.439)	.25		
	365	44.0	56.4	0.608 (0.235-1.577)	.31		

^a^Adjusted for age, ejection fraction <50%, left atrial enlargement, and amiodarone use post-CV

**Figure 4:**
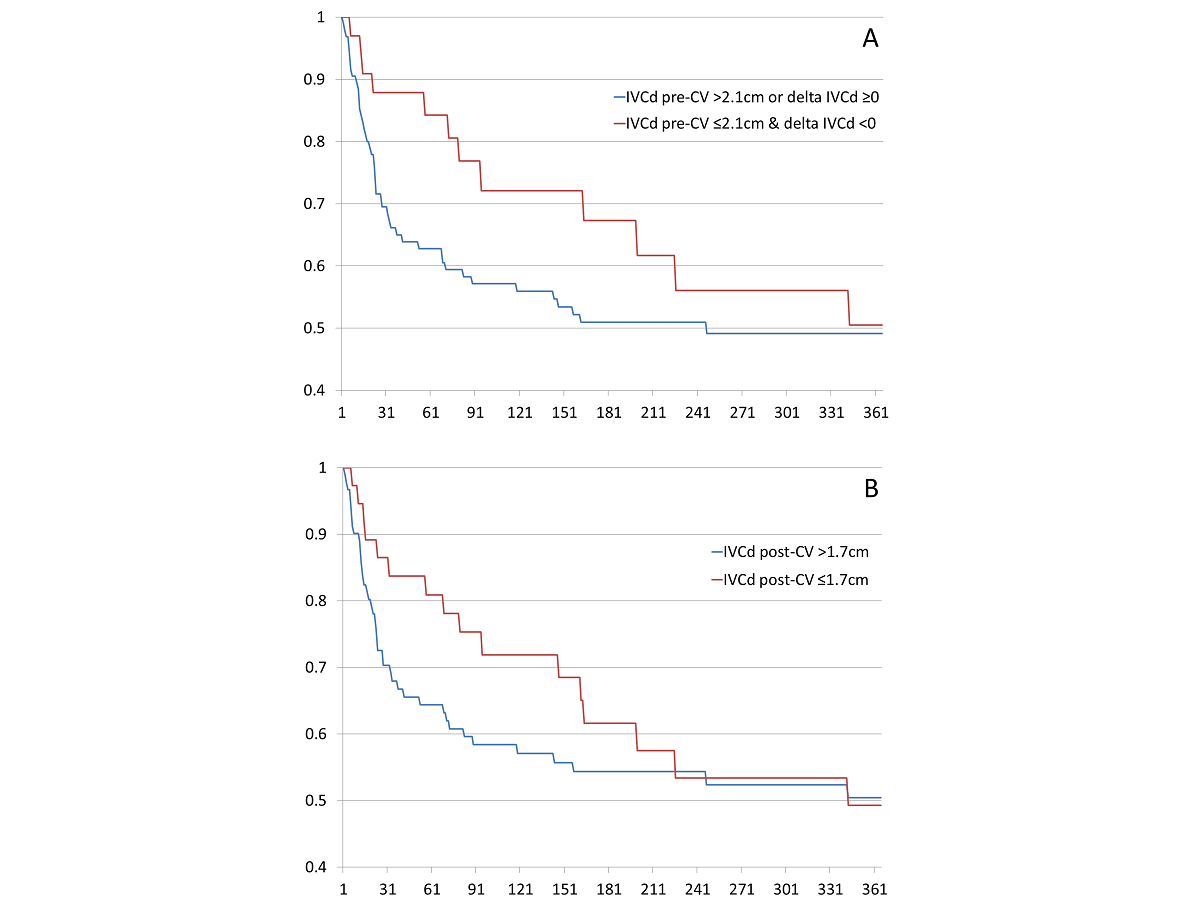
Handheld ultrasound.

At 90 days IVCd <1.7 cm post-CV was associated with a lower rate of AF/AF recurrence which was statistically significant after statistical adjustment, as noted above (unadjusted OR 0.435, 95% CI 0.182-1.039, *P*=.061; adjusted OR 0.344, 95% CI 0.124-0.958, *P*=.04). No significant association was seen with AF/AF recurrence at 180 days or 365 days.

Kaplan Meier curves were constructed to examine freedom from recurrence of AF/AF during follow-up (Figure 4). There were no significant differences overall in freedom from AF/AF after one year of follow-up between patients with the combination of IVCd pre-CV <2.1 cm and delta IVCd <0 compared to those without this combination of parameters (*P*=.10 by log-rank test), or between patients with IVCd post-CV <1.7 cm compared to those with IVCd post CV >1.7 cm (*P*=.21 by log-rank test). However, the curves diverged in the initial 30-90 days, before converging with longer-term follow-up.

No significant associations were found between AF/AF recurrence at any time and low, medium, or high RAP estimates pre-CV, high RAP estimate post-CV, or delta IVCd <0 in either bivariate or multivariate models. A low estimated RAP post-CV was associated with a lower likelihood of AF/AF recurrence at 30 days (OR 0.441, 95% CI 0.196-0.993, *P*=.05), and the association remained significant after adjustment (OR 0.393, 95% CI 0.11600.966, *P*=.04) but was not significant at the 90, 180, or 365-day time points (data not shown).

## Discussion

To our knowledge, this is the first study to evaluate measurements of IVC with HHU in the setting of AF/AF and CV. The key findings of this report are two-fold. First, we found that IVCd was larger and inspiratory collapsibility was less common in the presence of AF/AF compared to SR. These findings not only demonstrate that AF/AF influences the size and dynamics of the IVC, but also that HHU-derived measurements of the IVC (easily obtained anatomic measurements) are reflective of dynamic changes in physiology. These results suggest that changes in cardiovascular hemodynamics can be ascertained and quantified with a noninvasive handheld tool to potentially inform clinical decision support at the point of care.

Second, in our cohort, we found that a normal sized IVC (<2.1 cm) that becomes smaller following CV from AF/AF to SR, and a small IVC (<1.7 cm) following CV from AF/AF to SR are associated with decreased risk of early AF/AF recurrence at 30 or 90 days, even after adjustment for established clinical risk predictors of recurrence. In our study, these IVC parameters did not predict long-term risk of AF/AF recurrence beyond 90 days, suggesting that the factors mediating AF/AF recurrence evolve over time or that our cohort was insufficiently sized to make longer-term assessments of AF/AF recurrence. Further study may better differentiate potential subsets of patients in whom IVC parameters may be predictive of long-term outcomes.

Previously published data have demonstrated that atrial pressure is directly and immediately influenced by atrial rhythm. The first report of this phenomenon was published nearly half a century ago, when invasive measurements of RAP were shown to decrease immediately following CV from AF to SR [14]. A more contemporary study in an animal model also demonstrated higher atrial pressures in AF compared to SR [15]. Importantly, two recently published studies of patients undergoing catheter ablation of AF have demonstrated invasively measured correlates consistent with the findings from our study [10,11]. The first study showed a small but significant decrease in left atrial pressure following restoration of SR, with higher left atrial pressure in SR predicting risk of AF recurrence [10]. More recently, Kishima et al reported that an increase in left atrial pressure after CV was independently predictive of AF recurrence [11]. Our study represents the noninvasive corollary of these findings. We showed that noninvasive estimates of RAP decrease immediately following restoration of SR. We also showed that in our cohort IVC parameters reflective of a low RAP in SR, and a decrease in RAP after CV, predict freedom from AF/AF recurrence at 30 and 90 days.

A growing body of evidence is accumulating in support of the use of HHU as an adjunctive tool for bedside diagnoses, with superior and additive diagnostic performance compared to a physical examination alone [16]. Increasing attention is now being placed on the use of HHU to improve clinical decision support and risk prediction at the point of care. Recently published data suggest that serial monitoring of IVC size predicts risk of hospitalization in stable outpatients with heart failure [17,18]. The findings of the present study expand the possible roles of HHU even further to possible risk prediction in AF and use of HHU at the point of care during CV.

It is noteworthy that amiodarone use was associated with increased risk of AF/AF recurrence. This unexpected finding likely reflects the observational design of this study and suggests that the prescribing physicians may have anticipated a higher rate of recurrence in these patients. Nevertheless, after adjustment for amiodarone use, as well as previously described predictors of AF/AF recurrence (age, ejection fraction <50%, and left atrial enlargement) in our multivariate model, the HHU-derived measurements remained significant predictors of AF/AF recurrence in our cohort.

There are several important limitations to this study. First, because no intervention strategy was studied, these observational data can only be considered hypothesis-generating. An intervention trial would be needed to investigate whether a higher risk of recurrence indicated by IVC measurements at the time of CV might be mitigated by a change in treatment strategy. Second, AF and atrial flutter were grouped together for both patient enrollment and outcomes. The small number of patients with atrial flutter in this study precluded detailed assessment of whether the prognostic potential of IVC measurements applies equally to both AF and atrial flutter. In addition, the timing of IVC measurements (eg, pre- vs post-CV) was not blinded to the investigators acquiring them, introducing a potential for bias in measurement. Although the IVCd measurements were purely quantitative, in order to prioritize ease of acquisition, the assessment of IVC collapsibility was only performed as a visual estimate, which is a qualitative metric that is more vulnerable to the influence of bias. In addition, follow-up and ascertainment of echocardiographic information and past medical history were limited to review of the electronic medical record, and there was no requirement for concurrent echocardiogram at the time of enrollment in the study, nor any assessment of volume status at the time of CV. Therefore, although no significant associations were observed between echo-derived parameters and AF recurrence, the study was not designed to optimally compare the efficacy of established echo-derived or heart failure-based risk predictors of AF with HHU-derived IVC measurements. Another limitation of the study design is that invasive measurement of the RAP was not performed. However, ultrasound measurement of the IVC is well established as a correlate of invasive RAP measurement [19], and this study highlights the ability to gain potentially important prognostic information in a rapid, noninvasive fashion. Lastly, the hemodynamic effects of the use of propofol on RAP and IVCd cannot be reliably determined as meaningful from our cohort.

The duration of AF/AF at the time of CV was not rigorously recorded in this study. One could hypothesize that atrial remodeling and fibrosis from longstanding AF/AF would be associated with ineffective atrial contraction post-CV, and could influence IVC behavior in the early post-CV period. Atrial remodeling could mediate an association between larger IVCd post-CV and more frequent AF/AF recurrence. Further study inclusive of AF/AF duration and markers of atrial fibrosis could help to explore this potential connection.

## Abbreviations

AF: atrial fibrillation
AF/AF: atrial fibrillation or atrial flutter
CV: cardioversion
HHU: handheld ultrasound
IVC: inferior vena cava
IVCd: maximum inferior vena cava diameter
OR: odds ratio
RAP: right atrial pressure
SR: sinus rhythm
